# Comparison of pediatric and adult medullary thyroid carcinoma based on SEER program

**DOI:** 10.1038/s41598-020-70439-7

**Published:** 2020-08-06

**Authors:** Zhuang Zhao, Xiang-dang Yin, Xu-he Zhang, Zhi-wen Li, Dun-wei Wang

**Affiliations:** 1 Department of Anesthesiology, First Hospital of Jilin University, Changchun, 130021 Jilin China; 2 Department of Thyroidhyroid Surgery, Jilin Cancer Hospital, Changchun, 130012 Jilin China; 3 Department of Head and Neck Surgery, Jilin Cancer Hospital, Changchun, 130012 Jilin China

**Keywords:** Cancer, Oncology, Risk factors

## Abstract

To compare the clinicopathological characteristics and survival outcomes of children and adult diagnosed with medullary thyroid carcinoma (MTC). MTC patients were extracted from the Surveillance, Epidemiology and End Results (SEER) database from 1998 to 2016, followed by stratification into pediatric (< 20 years) or adult (≥ 20 years) groups. In total, 2,197 patients (110 pediatric and 2087 adult) with MTC were identified. Pediatric patients were more likely to have localized stage (70.0% vs. 51.6%), negative regional nodes (48.2% vs. 30.8%) and receive total/subtotal thyroidectomy surgery (97.3% vs. 85.3%). Moreover, CSS and OS rates were significantly higher in pediatric patients (both *P* < 0.001). Multivariable Cox regression analysis revealed that adult patients were significantly correlated with worse CSS and OS rates [(CSS: HR 11.60, 95% CI 1.62–83.02, *P* = 0.015); (OS: HR 5.63, 95% CI 2.08–15.25, *P* = 0.001)]. Further stratified analysis indicated that pediatric group might have significant better CSS and OS for patients with more advanced stage. Patients in the pediatric group were more likely to have earlier stage. Moreover, the prognosis of pediatric MTC patients was significantly better than that in adult patients.

## Introduction

Thyroid carcinoma is the most prevalent cancer of the endocrine system, accounting for about 1–3% of all human malignancies^1^. Medullary thyroid cancer (MTC) accounts for less than 5% of all types of thyroid cancers^2,3^. Compared to other types of thyroid cancer, MTC is more likely to present with more advanced and aggressive disease^4^. The incidence of pediatric MTC in pediatric is extremely low, with an estimated incidence of 0.03 cases per 100,000 population per year^5,6^. Due to the extremely low incidence of pediatric MTC, most clinicians, even with experienced thyroid practice, are unfamiliar with the clinicopathological characteristics and prognosis of pediatric MTC in comparison with other common thyroid cancers. Thus, it is necessary to highlight the awareness of MTC^7^. In consideration of the different clinicopathological characteristics and prognosis of MTC in children and adults, it is necessary to compare and analyze between the two groups^8^.

Currently, the therapeutic regimen and survival outcomes of MTC have been evaluated only by a small number of studies with small sample size^9–11^. Therefore, clinical decision-making concerning the optimal management of pediatric MTC is still challenging, which is generally based on extrapolation from adult MTC^9^.

The National Institutes of Health (NIH)’ s Surveillance, Epidemiology and End Results (SEER) database, the largest and most authoritative cancer dataset in North America^12^, covers approximately 30% of the US population from several different geographic regions^13^, which provides valuable data to investigate rare malignancies^14–17^. Herein, relevant data were extracted from the SEER database to determine the epidemiology, therapeutic strategies, and survival outcomes between pediatric and adult MTC patients.

In the present study, we aimed at elucidating the differences among pediatric and adult MTC via the comparison of tumor characteristics, therapeutic regimens and survival outcomes by using SEER datasets. Our findings can hopefully provide guidance in clinical decision-making and future directions in clinical trials.

## Materials and methods

### Study population

The qualified subjects were selected and determined utilizing the tool of SEER*State v8.3.6 released on August 8, 2019. It includes 18 SEER regions during the period 1998–2016. The inclusion criteria were as following: (1) it should be histological confirmed MTC patients; (2) the diagnosis of MTC was in accordance with the International Classification of Disease for Oncology, Third Edition (ICD-O-3; coded as 8510/3 and 8345/3). The exclusion criteria were shown as follows: (1) patients had more than one primary malignancies; (2) patients with reported diagnosis source from autopsy or death certificate or only clinically diagnosed; (3) patients have mixed pathological type; (4) patients without certain important clinicopathological data, such as: race, SEER summary stage and surgical style; (5) patients died of unknown cause; (6) patients without prognostic data. The eligible patients were included as SEER primary cohort, who were subsequently categorized into pediatric (< 20 years) and adult patients (≥ 20 years)^5,18,19^.

### Covariates and endpoint

We analyzed the patients’ characteristics according to the following factors: year of diagnosis (1998–2004, 2005–2010, 2011–2016), sex (male, female), race (white, black, or others), summary stage (localized, regional, distant), surgery(no surgery, lobectomy ,total/subtotal thyroidectomy), lymph node dissections (none or biopsy, 1–3 regional lymph nodes removed, ≥ 4 regional lymph nodes removed, unknown), regional nodes (negative, positive, and unknown);chemotherapy (no/unknown, yes), radiotherapy (no/unknown, yes). In terms of tumor staging, the SEER staging system was adopted due to its standardized definitions and consistent data recording. According to the SEER staging system, localized cancer was defined as tumors restricted to the organ where it began, without spreading, the definition of regional cancer was tumor spreading out of the primary site to the adjacent tissues or organs or lymph nodes, distant cancer was defined as tumors spreading beyond the primary site to distant lymph nodes or organs^20^.

The endpoint of this study was overall survival (OS) and cancer-specific survival (CSS). OS was defined as the duration from diagnosis to all-cause death and CSS was defined as the duration from diagnosis to MTC-caused death. According to the SEER 2018 submission database, the cut-off date was pre-determined until November 2018 (containing death data). Thus, the cut-off date was set at November 31, 2018.

### Statistical analyses

Chi-square test or Fisher’s exact test was adopted for categorical data to assess the differences between pediatric and adult groups. Kaplan–Meier (K–M) method was employed to estimate for univariate analysis, followed by log-rank test to determine the differences. Variables with *P* < 0.1 in univariate analysis were incorporated into multivariate Cox proportional hazard analysis. At the end, a stratified analysis was performed using the summary stage for those patients with log-rank test. SPSS software version 19.0 (SPSS Inc., Chicago, USA) was utilized for statistical analysis. The survival curves were generated by Graph Pad Prism 5. A two-sided *P* < 0.05 indicated statistical significance.

### Ethics statement

In order to obtain relevant data from the database, we signed the SEER Research Data Agreement (No.19817-Nov2018) and further searched for data according to the approved guidelines. The extracted data were publicly accessible and de-identified, and the data analysis was considered as non-human subjects by the Office for Human Research Protection, therefore, no approval was required from the institutional review board.

## Results

### Patient characteristics

**Figure 1:**
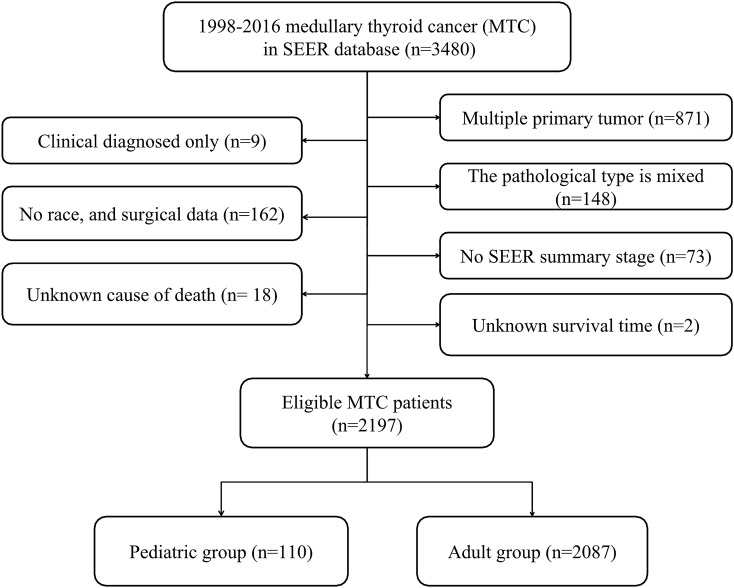
Flow chart for screening eligible patients.

In total, 3,480 MTC patients were included in the SEER database. Based on the inclusion and exclusion criteria, 2,197 eligible subjects were included in our research, with 110 pediatric patients, and 2087 adult patients. The detailed screening process was displayed in Fig. 1. The clinicopathological and therapeutic features in both groups were shown in Table 1. Except for year of diagnosis, sex, race and the extent of lymph node dissection, the rest variables were significantly different between the two groups (all *P* < 0.05). Compared with adult MTC, patients in the pediatric group had higher proportion of localized stage (70.0% vs. 51.6%) and negative regional nodes (48.2% vs. 30.8%). In terms of therapeutic modes, more pediatric patients received total/subtotal thyroidectomy surgery compared with adult patients (97.3% vs. 85.3%). Additionally, less pediatric patients had chemotherapy (0.9% vs. 5.7%) and radiotherapy (2.7% vs. 14.9%).

**Table 1:** Baseline characteristics of MTC patients included in this study (n = 2,179).

Characteristics	Pediatric (N = 110)	Adult (N = 2087)	*P*-value
**Year of diagnosis**			0.270
1998–2004	21 (19.1%)	540 (25.9%)	
2005–2010	40 (36.3%)	720 (34.5%)	
2011–2016	49 (44.6%)	827 (39.6%)	
**Sex**			0.707
Male	47 (42.7%)	854 (40.9%)	
Female	63 (57.3%)	1,233 (59.1%)	
**Race**			0.577
White	90 (81.8%)	1773 (85.0%)	
Black	11 (10.0%)	177 (8.5%)	
Other	9 (8.2%)	137 (6.5%)	
**Summary stage**			< 0.001
Localized	77 (70.0%)	1,077 (51.6%)	
Regional	28 (25.5%)	715 (34.3%)	
Distant	5 (4.55%)	295 (14.1%)	
**Surgery**			< 0.001
No surgery	0 (0.0%)	153 (7.3%)	
Lobectomy	3 (2.7%)	155 (7.4%)	
Total/subtotal thyroidectomy	107 (97.3%)	1779 (85.3%)	
**Lymph node dissection**			0.223
None or biopsy	21 (19.1%)	513 (24.6%)	
1–3 Regional lymph nodes	18 (16.4%)	223 (10.7%)	
≥ 4 Regional lymph nodes	51 (46.4%)	963 (46.1%)	
Unknown	20 (18.1%)	388 (18.6%)	
**Regional nodes**			< 0.001
Negative	53 (48.2%)	642 (30.8%)	
Positive	30 (27.3%)	852 (40.8%)	
Unknown	27 (24.5%)	593 (28.4%)	
**Chemotherapy**			0.028
No/unknown	109 (99.1%)	1968 (94.3%)	
Yes	1 (0.9%)	119 (5.7%)	
**Radiotherapy**			< 0.001
No/unknown	107 (97.3%)	1775 (85.1%)	
Yes	3 (2.7%)	312 (14.9%)	

### Survival analysis of all population

**Figure 2:**
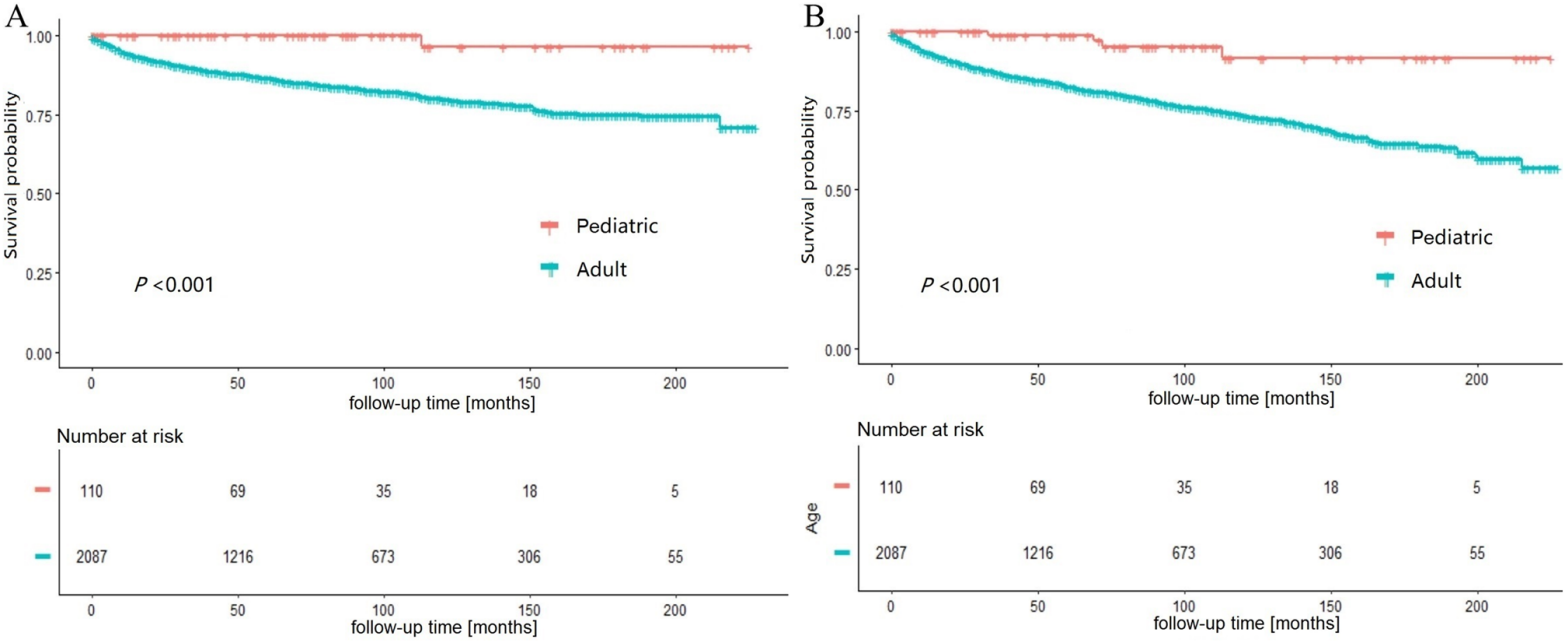
Kaplan–Meier curves for cancer-specific survival (**A**) and overall survival (**B**) in pediatric and adult groups.

The median follow-up duration was 65 months (0–227 months). The 3- , 5- and 10-year CSS rates were significantly higher in pediatric patients than in adult patients (100%, 100% and 96.30% vs. 89.21%, 86.23% and 79.62% respectively; *P* < 0.001) (Fig. 2A). Similarly, the 3-, 5- and 10-year OS rates were significantly higher in pediatric patients than adult patients (98.80%, 98.80% and 91.74% vs. 86.97%, 82.56% and 73.19% respectively; *P* < 0.001) (Fig. 2B).The CSS and OS curves were displayed in Fig. 2.

### Univariate and multivariate analyses

Univariate analysis revealed that year of diagnosis, age, sex, summary stage, surgery, lymph node dissections, the status of regional nodes, chemotherapy, and radiotherapy were risk factors for CSS (*P* < 0.05). All the above variables were incorporated into the multivariate Cox analysis, which showed adult group was associated with significantly worse CSS rates (hazard ratio [HR] 11.60, 95% CI 1.62–83.02, *P* = 0.015) compared with the pediatric group after the adjustment of confounding factors. Similarly, multivariate analysis on OS suggested that adult patients were related to significantly worse survival than pediatric patients (HR 5.63, 95% CI 2.08–15.25, *P* = 0.001). In addition, multivariate analysis further revealed that advanced summary stage, no surgery and radiotherapy were also independent unfavorable prognostic factors of CSS and OS. The detailed findings of univariate and multivariate analysis were available in Table 2.

**Table 2:** Univariate and multivariate analyses of cancer special survival (CSS) and overall survival (OS) for 2,197 patients with MTC.

Variables	CSS			OS		
	Univariate analysis	Multivariate analysis		Univariate analysis	Multivariate analysis	
	*P* value	HR (95%CI)	*P* value	*P* value	HR (95%CI)	*P* value
**Year of diagnosis**	0.010		0.210	0.016		0.174
1998–2004		Reference				
2005–2010		0.78(0.56,1.10)	0.164		0.84 (0.63,1.13)	0.246
2011–2016		0.71(0.48,1.05)	0.084		0.72(0.51,1.02)	0.061
**Age**	< 0.001			< 0.001		
Pediatric		Reference			Reference	
Adult		11.60(1.62,83.02)	0.015		5.63 (2.08,15.25)	0.001
**Sex**	< 0.001			< 0.001		
Male		Reference			Reference	
Female		1.11(0.88,1.40)	0.370		0.99(0.81,1.20)	0.885
**Race**	0.152		NI	0.063		0.288
White					Reference	
Black					1.27(0.93,1.72)	0.132
Other					0.93(0.64,1.37)	0.724
**Summary stage**	< 0.001		< 0.001	< 0.001		< 0.001
Localized		Reference			Reference	
Regional		6.29(3.70,10.69)	< 0.001		3.04(2.05,4.49)	< 0.001
Distant		25.21(14.76,43.04)	< 0.001	< 0.001	9.66(6.49,14.38)	< 0.001
**Surgery**	< 0.001		< 0.001			< 0.001
No surgery		Reference			Reference	
Lobectomy		0.56(0.32,0.98)	0.042	< 0.001	0.49(0.30,0.79)	0.003
Total thyroidectomy		0.43(0.29,0.65)	< 0.001		0.43(0.30.0.61)	< 0.001
**LN dissection**	< 0.001		0.242	< 0.001		0.247
None or Biopsy		Reference			Reference	
1–3 regional LN		1.90(0.64,1.83)	0.749		1.19(0.76.1.85)	0.449
≥ 4 regional LN		0.75(0.49,1.13)	0.170		0.83(0.58,1.19)	0.315
Unknown		1.01(0.65,1.56)	0.969		1.05(0.73,1.51)	0.804
**Regional nodes**	< 0.001		0.091	< 0.001		0.017
Negative		Reference			Reference	
Positive		2.25(0.74,6.84)	0.097		1.41(0.91,2.17)	0.122
Unknown		2.04(1.07,3.88)	0.031		1.58(1.28,1.95)	0.005
**Chemotherapy**	< 0.001			< 0.001		
No		Reference			Reference	
Yes		1.25(0.91,1.72)	0.161		1.15 (0.85,1.56)	0.357
**Radiotherapy**	< 0.001			< 0.001		
No		Reference			Reference	
Yes		1.66(1.32,2.10)	< 0.001		1.15 (0.85,1.56)	< 0.001

*CSS* cancer‐specific survival, *OS* overall survival, *NI* not included in the multivariate survival analysis, *LN* lymph nodes.

### Stratified survival analysis based on SEER summary stage

**Figure 3:**
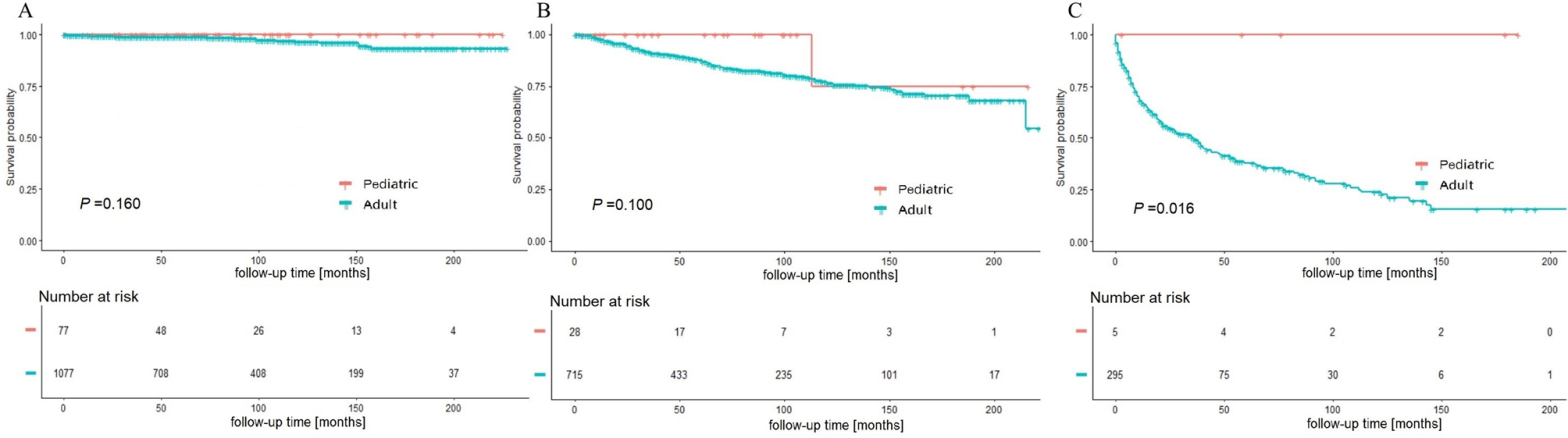
Cancer-specific survival of (**A**) localized stage, (**B**) regional stage, and (**C**) distant stage MTC are shown, stratified by age group.

**Figure 4:**
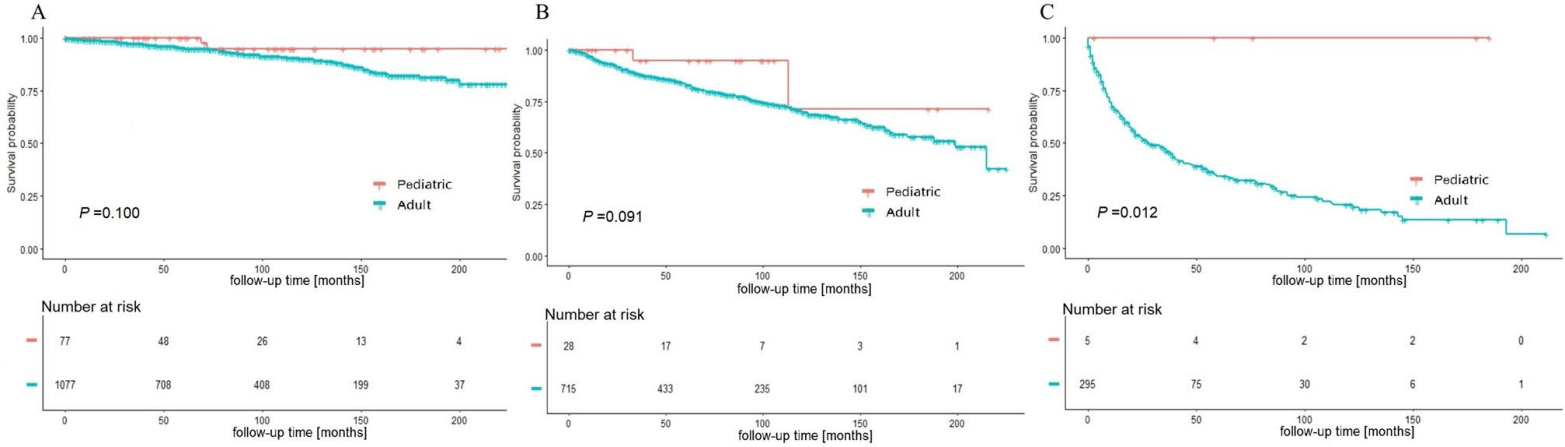
Overall survival of (**A**) localized stage, (**B**) regional stage, and (**C**) distant stage MTC are shown, stratified by age group.

For better understanding of the survival advantage of pediatric MTC patients, stratified analysis based on SEER summary stage was conducted to analyze the impacts of age on survival (Figs. 3, 4). The K–M plots implicated that pediatric group had significant CSS and OS benefits for patients at advanced stage (*P* = 0.016 and *P* = 0.012, respectively). However, the differences in localized and regional stages between the two groups were not significant.

## Discussion

The analysis presented in this study compares the outcome of 110 pediatric with 2087 adult MTC patients in the SEER database. Pediatric patients were found to be with earlier stage. In addition, patients in the pediatric group showed significantly better CSS and OS rates than adult.

There has always been a debate about the age onset of children and adults division. The majority of thyroid cancers in children were defined as less than 20^5,18,19^ or 18 years old^21,22^. We have systematically referred to a lot of previous studies, and selected some more relevant literatures with our purpose^5,18,19,23^. In the end, we define pediatric MTC as being < 20 years old.

About 1,200 new MTC cases are diagnosed every year in the US^24^. The incidence of pediatric MTC is approximately 0.03 per 100,000 annually^5,6^. MTC occurs either sporadically or in an inherited autosomal dominant manner. Sporadic MTC accounts for 65–75% of adult patients with MTC; while sporadic MTC is barely detected in children, and most pediatric MTC is hereditary^5^. The prognosis of pediatric MTC is extremely satisfactory, with the 5-year OS of 95% and 15-year OS of 86%^5^, which is similar to the results of our research.

Because of its rarity, there are few reports on the prognosis of pediatric MTC. Nevertheless, some studies have found that age affects the prognosis of MTC patients^25,26^. Roman et al. even believed that age is the most important prognostic factor of MTC, second only to tumor stage^25^. Our study not only found the significantly better prognosis in pediatric patients than adult patients, but also revealed that the death risk in adult patients was 11 times higher than that of pediatric patients on CSS, and 5 times higher on OS.

Similarly, surgical resection also plays a vital role in pediatric MTC. The standard treatment for MTC is surgical removal of all thyroid tissue including the posterior capsule^27,28^. There are not many therapeutic options for MTC other than surgery. Radiotherapy or chemotherapy is not recommended for pediatric MTC^29^. Targeted molecular therapy plays an effective role in treating patients with metastatic MTC. The majority of familial MTCs have oncogene rearranged during transfection (RET) mutation^30^. Meanwhile, somatic RET mutation is detected in 65% of sporadic MTC^31,32^. In addition, RAS mutation or vascular endothelial growth factor receptor (VEGFR) up-regulation making them suitable for targeted therapy. Meanwhile, these gene mutations have practical value for following-up of pediatric patients^30^. Tyrosine kinase inhibitors (TKI), small molecules, can suppress the activity of tyrosine kinases, which could otherwise trigger invasion, angiogenesis, proliferation, as well as metastases^33^. Therefore, TKIs, especially cabozantinib and vandetanib, have been accumulatively adopted to manage advanced MTC^34,35^.

There are certain limitations in our research. Firstly, As a retrospective research based on SEER database, the intrinsic selection biases exists in this study^15,17^. Secondly, not all data are available from the SEER database, such as quality-of-life, family history of cancer, molecular-genetic profiles, detailed information about chemotherapy and radiotherapy. Thirdly, because of the large time span of included patients, we could not use the unified AJCC stage, which reduces the practical value of this study. Fourthly, the number of pediatric MTC in advanced stage is small, only five cases. In this case, the stratified analysis used will cause a certain statistical bias, so it is necessary to be cautious in interpreting the results. Finally, we also need to point out that for hereditary MTC; there are some patients with unilateral or bilateral pheochromocytoma. This part of MTC patients should be retained, could not be excluded as patients with a second primary tumor. Unfortunately, there is no detail information about the second or third primary cancer in our data, so we can not strictly screen and retain this part of patients. The above limitations might cause study bias and damage the power of analysis. Although it was better to obtain more details, we aimed to show the survival advantage in pediatric patients over adult patients. In this regard, we believed that the currently available data in the SEER database could serve our research objectives very well.

## Conclusion

In summary, pediatric MTC patients have earlier (or less advanced) stage than adult patients. Moreover, the prognosis of pediatric MTC patients is significantly better than that in adult patients, which also indicates that more active interventions should be performed in the treatment for pediatric patients. In consideration of the limitations in SEER datasets, our research might provide a new direction on MTC, to further attract attention to fully answer the questions based on delicately-designed prospective studies.
